# Heavy Metal Contamination and Health Risk Assessment in the Vicinity of a Tailing Pond in Guangdong, China

**DOI:** 10.3390/ijerph14121557

**Published:** 2017-12-12

**Authors:** Yaya Liang, Xiaoyun Yi, Zhi Dang, Qin Wang, Houmei Luo, Jie Tang

**Affiliations:** 1 School of Environment and Energy, South China University of Technology, 382 Waihuan East Road, Guangzhou 510006, China; liangyayalh@163.com (Y.L.); chzdang@scut.edu.cn (Z.D.); wq102@outlook.com (Q.W.); lhoumei@gxu.edu.cn (H.L.); 15727651980@163.com (J.T.); 2 The Key Lab of Pollution Control and Ecosystem Restoration in Industry Clusters, Ministry of Education, 382 Waihuan East Road, Guangzhou 510006, China

**Keywords:** tailing pond, heavy metals, water, soil, vegetables, rice, contamination, health risk

## Abstract

The purpose of this study was to assess heavy metal contamination and health risks for residents in the vicinity of a tailing pond in Guangdong, southern China. Water, soil, rice, and vegetable samples were collected from the area in the vicinity of the tailing pond. Results showed that surface water was just polluted by Ni and As, while groundwater was not contaminated by heavy metals. The concentrations of Pb, Zn, Cu, Cd, Ni, and As in the paddy soil exceeded the standard values but not those of Cr. In vegetable soils, the concentration of heavy metals was above the standard values except for Ni and As. Soil heavy metal concentrations generally decreased with increasing distance from the polluting source. Leafy vegetables were contaminated by Pb, Cr, Cd, and Ni, while the non-leafy vegetables were contaminated only by Cr. There was a significant difference in heavy metal concentrations between leafy vegetables and non-leafy vegetables. Almost all the rice was polluted by heavy metals. Diet was the most significant contributor to non-carcinogenic risk, which was significantly higher than the safe level of 1. The total cancer risk was also beyond the safe range (10^−6^–10^−4^). Results revealed that there is a risk of potential health problems to residents in the vicinity of the tailing pond.

## 1. Introduction

With the rapid development of China’s industrialization, the demand for mineral resources has progressively increased. It was reported that there are 26,000 small-sized mines and more than 9000 large and medium-sized mines in China [1]. However, mining exploitation and ore smelting are important sources of heavy metals that cause environmental pollution. Large amounts of tailings and wastewater are produced in the mining process, which leads to severe heavy metal contamination in the surrounding environment. In recent years, heavy metal pollution problems arising from mining have attracted increasing attention. Emissions of toxic heavy metals can contaminate surface water, groundwater, agricultural soils, and food crops, and they pose health risks to the population via different pathways [2,3,4].

Heavy metal pollution has become a serious problem in the vicinity of mining sites. There are toxic heavy metals in wastewater drained from tailing ponds. Using this wastewater for irrigation leads to heavy metal contamination of agricultural lands and food crops. Wastewater may contain various toxic heavy metals, including Pb, Zn, Cu, Cr, Cd, Ni, and As. Besides, the tailing pond can contaminate the groundwater surrounding the tailing pond. These heavy metals are deemed to threaten human health and pose a cancer risk to humans [5,6]. Heavy metals in soils can contaminate the environment and damage human health through various exposure pathways including direct ingestion, dermal contact, and inhalation. Metal uptake has been studied in several crops (spinach, clover, grape vines, shrubs, barley, and wheat) by considering the edible part of most plant species; thus, the risk of metals in edible parts of crops to humans should be a matter of concern [7]. The crops that contain heavy metals absorb toxic elements from contaminated soils through their roots, and leaves also can absorb noxious elements from particles deposited on their surfaces. As heavy metals in crops enter into the human body through the food chain and present health risks to humans, food consumption is also an important exposure pathway. Vitamin C, Fe, and other nutrients stored in the body are significantly decreased if humans consume food contaminated by Pb, Cd, As, and other toxic elements, leading to a decline in immunity, a deterioration of human functions, and even disabilities associated with malnutrition [2]. In order to estimate the risk quantifiably, we employed the health risk assessment model recommended by U.S. EPA [8]. The hazard index and cancer risk were used to signify non-carcinogenic effects and carcinogenic effects, respectively. Many researchers have adopted this method to estimate the health risk to humans [9,10,11]. It is reported that the total hazard indices of As, Pb, and Cd are greater than or close to the safety threshold of 1, which poses potential health problems to residents in the vicinity of the mining industry [12]. A researcher collected and analyzed samples of tailing, soil, water, and crop plants in the vicinity of the abandoned mine, and calculated hazard quotient values for the pathways of soil ingestion, crop ingestion, water ingestion, and water dermal contact; the total value was 16, which indicated that the mine has a tremendous health risk to the residents [13]. Since there is more than one pathway of heavy metals absorption, taking account of various exposure pathways in the process of health risk assessment is of significance for inhabitants in the vicinity of mining areas.

The main objectives of this work are: (1) to explain the content of Pb, Zn, Cu, Cr, Cd, Ni, and As in surface water and groundwater, as well as content variation in all surface water samples along the river, (2) to delineate the heavy metal concentrations in soil and content variation in different soil samples; (3) to determine the content of heavy metals in edible parts of vegetables and rice; and (4) to estimate the health risk to inhabitants produced by heavy metals via various exposure pathways.

## 2. Samples and Methods

### 2.1. Study Area

The study area, Bing village Pb-Zn tailing pond (24°23′10′′ N, 116°13′0′′ E) is located in Bing village, Meizhou city, Guangdong, Southern China. The tailing pond is located in northeast of Meizhou city; it is about 15 km away from Meizhou city and 7.6 km away from Bing village. This area has a subtropical monsoon climate with an annual average temperature of 21.3 °C and annual rainfall of 1528.5 mm. The tailing pond was built in 1975 and closed in 1992. The long-term shutdown and unmanned management of the tailing pond result in disrepair, blockage of drainage system, landslide of dam, and shortage of flood storage capacity, and the security classification of the tailing pond is dangerous. In July 2013, the dam collapsed due to typhoon named “Su Li” with heavy rainfall, which had a tremendous impact on the surrounding environment of reservoir area. The volume of tailings is about 38 × 10 ^4^ m^3^. The drainage from the pond runs off into streams and rivers, which are mainly used to irrigate farmland for field crops and vegetables. Meanwhile, the tailings exposed to weather are dry, and because of wind flow, the particles spread into the surrounding environment. Since there are many farmlands surrounding the tailing pond, the high concentration heavy metals in the crops planted in the farmland might likely result in adverse effects to the residents around this area. 

### 2.2. Sample Collection and Pre-Treatment

**Figure 1:**
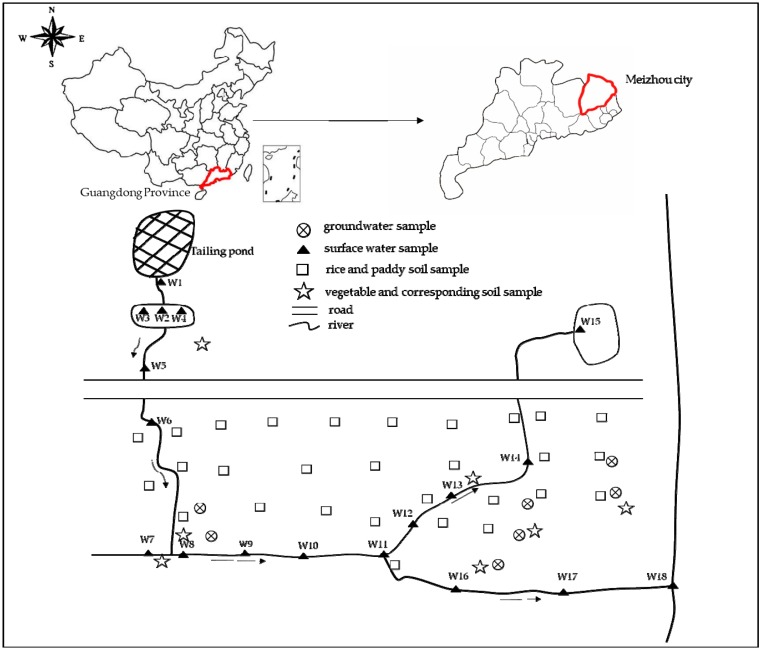
Sketch map of research area and sampling points in Guangdong Province, China.

The map of study area and sample point is shown in Figure 1. We collected 18 surface water samples from the river located downstream from the tailing pond, and the water is mainly used to irrigate the crops. In order to investigate the concentration of heavy metals in groundwater in the study area, we collected seven groundwater samples from wells in resident households. According to the investigation in the study area, the groundwater is not used for drinking purposes. Water samples were put in clean polyethylene bottles and the pH was determined immediately. All water samples were brought to the laboratory, acidified with HNO_3_, and stored at 4 °C before analysis. Crop samples (rice and vegetables) and corresponding rhizosphere soils (0–20 cm) were collected in March and July of 2017. The common vegetables and other crops grown in the study area are rice (*Oryza sativa* L.), leafy vegetables (including scallion (*Allium fistulosum*), garlic sprout (*Allium porrum*), cabbage (*Brassica oleracea*), water spinach (*Ipomoea aquatica* Forsskal), sweet potato leaves (*Ipomoea batatas* (L.) Lam.), Indian lettuce (*Lactuca sativa* var longifoliaf. Lam), cow soapwort (*Vaccaria segetalis* Garcke)), and non-leafy vegetables (including cowpea (*Phaseolus vulgaris*), eggplant (*Solanum melongena*), capsicum (*Capsicum annuum* Linn.), and okra (*Abelmoshus esculentus*)). In a sampling unit, rice and vegetables were collected using the plum blossom sampling method. The corresponding rooted soils were collected using the same method. All samples were put in clean plastic bags and transported to the laboratory for treatment as soon as possible. The vegetable samples were washed with tap water and rinsed with deionized water three times; the surface water was absorbed with filter paper, and the fresh weights (FW) were recorded. The fresh samples were dried at 75 °C to a constant weight in an oven; the dried samples were weighed again, and the dry weight (DW) was recorded to calculate water content. The dried samples were then ground into a fine powder which could pass the 60-mesh nylon sieve and were stored in plastic bags. The edible parts of rice samples were washed with deionized water, ground into fine powder which could pass the 20-mesh nylon sieve, and stored in plastic bags. The soil samples were air dried, crushed with a mortar, passed the 20-mesh and 100-mesh nylon sieve, respectively, and stored in a dryer until an analysis of soil properties and heavy metal concentrations could be performed. 

### 2.3. Analysis and Quality Control

Soil pH was measured in ultrapure water (1:2.5 *w/v*) after stirring for 4 h and centrifuging at 3000 r/min. The organic matter content of soils was measured using the potassium dichromate oxidation method [14]. The cation exchange capacity (CEC) was determined in BaCl_2_∙2H_2_O and MgSO_4_∙7H_2_O extracting solutions [15]. The soil samples were digested with a concentrated acid mixture (HCL, HNO_3_, HF, and HClO_4_). The edible parts of rice and vegetables were digested with HNO_3_ and HClO_4_ in a 5:1 ratio. The digested solutions were cooled to room temperature and transferred to 50 mL colorimetric tubes, made up to volume with deionized water, and stored in clean plastic containers at 4 °C before analysis. All samples were analyzed with inductively coupled plasma atomic emission spectroscopy (ICP-AES) (Agilent, Santa Clara, CA, USA) and inductively coupled plasma mass spectrometry (ICP-MS) (Agilent, Santa Clara, CA, USA). The standard reference materials of soil sample (GBW07405(GSS-5)) and celery sample (GBW10048(GSB-26)) were digested and analyzed along with samples for quality control. Simultaneously, reagent blank determinations were used to correct the instrument readings. Samples were analyzed in triplicate to ensure precision and accuracy in the procedure of analyses of heavy metals. 

All statistical analyses were performed with Microsoft Excel and SPSS 22.0 for Windows (SPSS Inc., Chicago, IL, USA). 

### 2.4. Risk Assessment Methods

Health risk is defined as the likelihood of harmful effects to human health as a result of environmental pollution. In the study, we employed the health risk assessment model generated by United States Environmental Protection Agency (U.S. EPA) to assess the human health risk of heavy metals to adults. This risk assessment method has been used by many researchers [16,17,18,19]. The steps of health risk assessment included hazard identification, dose-response assessment, exposure assessment, and risk characterization [8,20]. 

According to the actual investigation of the study area, human beings could be exposed to heavy metals via the following four main pathways: (1) direct ingestion of soil particles, (2) inhalation of soil particles from the air, (3) dermal absorption of soil particles, (4) diet of vegetables and rice. 

#### 2.4.1. Calculation of Heavy Metal Intake

Strictly speaking, human pollutant intake refers to the effective dose of pollutants that can enter the body’s blood and effect on human tissue and organs. However, limited by the level of study, as well as taking conservative principles into account, the calculation of intake is expressed in the amount of pollutants absorbed by the body per body weight in unit time based on the potential dose in general. Heavy metal intake is just the chronic daily intake dose (CDI, mg/kg/day) of noxious substances during the exposure period. The calculation methods of CDI vary across different exposure pathways, which are shown in Table 1. The definition and value of exposure parameters are listed in Table 2.

#### 2.4.2. Human Health Risk Assessment

Health risks caused by different contaminants that enter the body through diverse exposure pathways are divided into carcinogenic risk and non-carcinogenic risk. 

Carcinogenic risk refers to the incremental probability of an individual developing any kind of cancer in a lifetime as a result of exposure to carcinogens. Carcinogenic risk can be evaluated by the following linear equation:

Cancer risk = CDI × SF(1)

where cancer risk is a unitless probability of an individual developing cancer, CDI is chronic daily intake dose of carcinogens (mg/kg/day), and SF is the carcinogenicity slope factor (mg/kg/day). The slope factor (SF) converts estimated daily intake averaged over a lifetime of exposure directly to incremental risk of an individual developing cancer [8]. Slope factors of As for ingestion, dermal, and inhalation were 1.5, 3.66, and 15.1 mg/kg/day, respectively, and the SF for inhalation of Cr, Cd, and Ni were 42, 6.3, and 0.84 mg/kg/day, respectively [13,25]. The cancer risk caused by a variety of carcinogens is the sum of carcinogenic risk of individual carcinogens in the possible exposure pathways, which is the total cancer risk (R). According to the U.S. EPA, the value of cancer risk in the range of 10^−6^ to 10^−4^ is an acceptable or tolerable risk, a risk of less than 10^−6^ can be ignored, and a risk exceeding 10^−4^ is considered to unacceptable.

Non-cancer risk is evaluated by comparing an exposure level over a specified time period (e.g., lifetime), with a reference dose derived for a similar exposure period. The non-cancer risk can be characterized as a hazard quotient (HQ). The hazard quotient is the ratio of chronic daily intake (CDI) and chronic reference dose (RFD). The equation is HQ = CDI/RFD. The oral reference doses were 3.5 × 10^−3^, 0.3, 4 × 10^−2^, 3 × 10^−3^, 1 × 10^−3^, 2 × 10^−2^, and 3 × 10^−4^ mg/kg/day for Pb, Zn, Cu, Cr, Cd, Ni, and As, respectively; dermal reference doses were based on 5.25 × 10^−4^, 6 × 10^−2^, 1.2 × 10^−2^, 6 × 10^−5^, 1 × 10^−5^, 5.4 × 10^−3^, and 1.23 × 10^−4^ mg/kg/day for Pb, Zn, Cu, Cr, Cd, Ni, and As, respectively, and the inhalation reference doses were 3.52 × 10^−3^, 3.00 × 10^−1^, 4.02 × 10^−2^, 2.86 × 10^−5^, 2.4 × 10^−6^, 2.06 × 10^−2^, and 3.01 × 10^−4^ mg/kg/day for Pb, Zn, Cu, Cr, Cd, Ni, and As, respectively [25,26,27]. The Hazard Index (HI) is used to assess the overall non-carcinogenic risk posed by more than one toxicant. For multiple hazardous substances, the hazard index is the sum of HQ of the individual toxic element. If the value of HQ or HI is less than one, it is unlikely to create adverse health effects for exposed populations. If the value of HQ or HI exceeds one, it is not in the acceptable range, and the greater the value, the greater the probability of the occurrence of adverse health effects.

## 3. Results and Discussion

### 3.1. Concentration of Heavy Metals in Surface Water and Groundwater

Eighteen surface water samples, which were taken along the downstream of river, and seven groundwater samples from wells were collected to analyze the heavy metal contamination in water. The total concentrations of heavy metals in surface water and groundwater are presented in Table 3. The surface water was slightly alkaline, while the groundwater was slightly acidic. The average concentrations of Pb, Zn, Cu, Cr, Ni, and As were 4.33, 269.90, 2.40, 1.69, 1.04, 11.40, and 24.62 μg/L, respectively. Zn has the highest recorded mean concentration and Cd has the lowest mean concentration. When compared with the permissible level of Grad V of Environmental Quality Standard for Surface Water [28], the concentrations of Pb, Zn, Cu, Cr, and Cd in surface water were far below the permissible level, while Ni and As exceeded this level by 27.8% and 5.6%, respectively. For groundwater, the concentrations of Pb, Zn, Cu, Cr, Cd, Ni, and As were 7.71, 102.66, 13.63, 3.69, 0.45, 3.56, and 1.36 μg/L, respectively. The heavy metal concentrations in groundwater were trace amounts below the value of Grad III of Environmental Quality Standard for Groundwater [29]. Based on the above analysis, the surface water was contaminated slightly by Ni and As, and the groundwater was not contaminated. The low concentrations of heavy metals in the surface water may be due to dilution of heavy metals in water medium, but the continuous application of surface water for irrigation resulted in accumulation of heavy metals into the soil.

**Figure 2:**
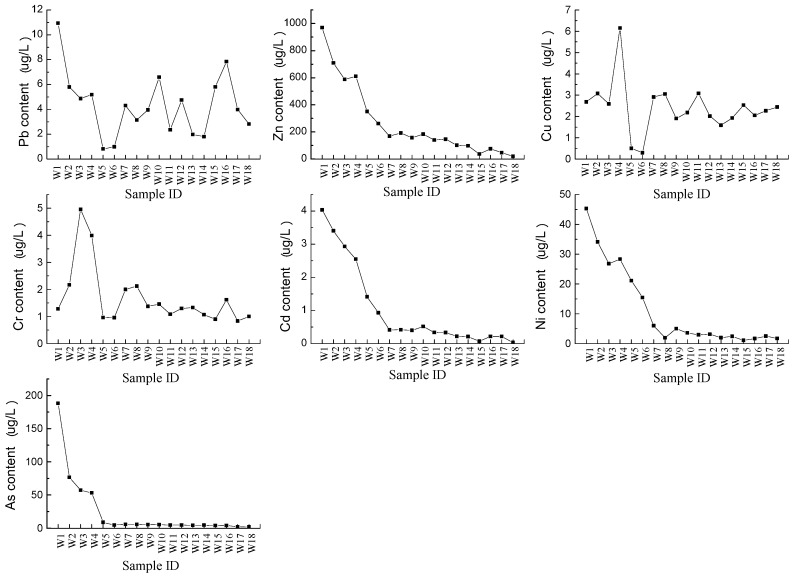
Heavy metal concentrations in surface water.

Heavy metal concentrations in different sampling sites are presented in Figure 2. In all the surface samples, Zn had the highest recorded concentration, which was about 969.74 μg/L in site “W1”, while Cd had the lowest concentration, which was around 0.04 μg/L in site “W18”. On the whole, the content of heavy metals varied in different sampling sites and different heavy metals had different variation characteristics. A similar variation appeared in the concentrations of Zn, Cd, Ni, and As; the concentrations decreased along the downstream of river, and the highest recorded concentrations existed in site “W1”. The concentration of As in site “W1”, which was closest to tailing pond, exceeded the permissible level, and the concentration declined dramatically from site “W1” to site “W5”, with the concentration of samples beyond site “W5” becoming stabilized. The concentrations of Zn, Cd, and Ni in all sampling sites varied with the same regularity; the concentration from site “W1” to site “W7” generally decreased, and sampling site “W8” was the beginning of relatively stable point for concentration. Zn is subjected to the processes of sorption mainly on oxy-hydroxides of iron and manganese and clayey minerals. Thus, the natural environmental conditions prevent the transportation of Zn as dissolved in the water and instead promotes their accumulation in riverine sediments [30]. Thus, the Zn content decreased along the river. The behavior of Cd is strongly related to pH; the solubility of Cd increases at pH < 6, and undergoes a slight adsorption by colloids of the soil, hydroxides, or organic matter. At pH > 6, cadmium is absorbed by the solid phase or precipitates, the absorbed Cd is not easily mobilized [31], and the pH of surface water we collected is greater than 6; this may be the cause of the diminishment in Cd concentration along the river. The mobility of As is rather limited and is hardly leached by the river due to its adsorption on clay and hydroxides [32], which caused the decline of As concentration along the river. Similar to Zn, Cd, and As, we think the mobility of Ni is low, and it is easily absorbed by sediments. Based on the above analysis, the concentrations of Zn, Cd, Ni, and As showed a downward trend totally. 

However, there was no regular fluctuation to concentrations of Pb, Cu, and Cr for all sampling sites, and the concentration of Pb fluctuated considerably, indicating that there was no significant rule for the concentration variation of Pb along the river. The highest concentrations of Pb, Cu, and Cr were in sites “W1”, “W4”, and “W3”, respectively. Sites “W1”, “W2”, “W3”, and “W4” were downstream and closer to the tailing pond than other sites, and their concentrations were relatively high. Normally, the randomness of heavy metal contents is relatively large and the distribution of heavy metal contents in water is irregular; heavy metal concentrations in water are affected by many factors, including particulate matter content, pH, chemical oxygen demand, and hydrodynamic conditions [33]. Consequently, it is normal to have some fluctuations in some heavy metal concentrations; different heavy metals present different distribution patterns in the surface water.

### 3.2. Heavy Metals in Soils

#### 3.2.1. Physicochemical Parameters

The main physicochemical parameters measured for all soil samples were as follows: (I) the pH value was varied: the soils were slightly acidic, with mean pH values of 6.25 and 6.51, and a range of 5.47–7.46 and 4.70–7.40 for paddy and vegetable soils, respectively. (II) Organic matter contents of paddy soils were equal to those of vegetable soils, with an OM range of 2.74–8.66% and 2.07–8.68% for paddy and vegetable soils, respectively. (III) The average CEC values in paddy and vegetable soils were 11.6 and 11.9 cmol/kg, with a range of 4.8–19.0 cmol/kg and 5.7–20.3 cmol/kg, respectively.

#### 3.2.2. Heavy Metal Levels in Soil

**Figure 3:**
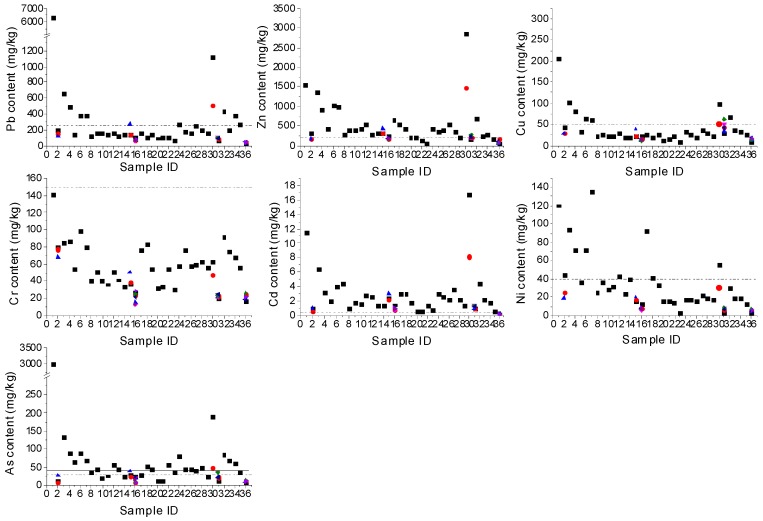
Heavy metal concentrations in all soil samples. Notes: Sample ID 1–36 were compiled with increasing distance from the tailing pond, and sample from ID 1 stand for the tailing sample; since some various vegetable soils were collected in a smallish area, the sample ID of these vegetable soils sampled from a smallish area are represented by the same sample ID, we use different colors to represent these vegetable soils; reference lines (dash) are Grad II values of Environmental Quality Standards for Soils in China (GB15618-1995), while reference line (solid) for As is the standard value of paddy soil.

Heavy metal concentrations in tailing, paddy soil, and vegetable soil are presented in Table 4. The elevated concentrations of Pb, Zn, Cu, Cd, Ni, and As in tailing exceeded the standard values, while the Cr content was less than standard value. The mean concentrations of Pb, Zn, Cu, Cd, Ni, and As were 25.4, 7.8, 4.1, 38.0, 2.9, and 75.5 times, respectively, above Grade II of Environmental Quality Standards for Soils of China [34]. Mean concentrations of Pb (245.6 mg/kg), Zn (491.0 mg/kg), Cu (35.6 mg/kg), Cr (59.8 mg/kg), Cd (2.6 mg/kg), Ni (37.1 mg/kg), and As (54.8 mg/kg) in paddy soils were slightly higher than those in vegetable soils. Average concentrations of Pb, Zn, Cu, Cr, Cd, Ni, and As were about 7, 10, 2, 1, 46, 3, and 6 times higher in paddy soils than Background Values of Soils in Guangdong Province [35]; the Cr and Ni concentrations in vegetable soils were less than the background values, while the mean content of Pb, Zn, Cu, Cd, and As were 3.8, 6.8, 1.6, 31.5, and 2.8 times higher in vegetable soils, respectively, illustrating that the external input of these heavy metals had a significant influence on the accumulation of elements in soils, being especially apparent for Cd. Compared to the values of Grade II of Environmental Quality Standards for Soils of China [30], almost all heavy metal concentrations exceeded the standard values, except for Cr (Figure 3). The percentages by which the paddy soils and vegetable soils exceeded the standard values were 27.6%, 89.7%, 17.2%, 0.0%, 100.0%, 24.1%, and 82.8%, and 10.3%, 27.6%, 3.5%, 0.0%, 75.9%, 3.5%, and 10.3% for Pb, Zn, Cu, Cr, Cd, Ni, and As, respectively, indicating that the paddy soils exceeded the standard values more than the vegetable soils. Figure 3 shows that the total percentage in excess of all soil samples decreased in the order of Cd > Zn > As > Pb > Ni > Cu > Cr. The concentrations of heavy metals varied with the distance from the tailing pond. Generally, it showed that the heavy metal concentrations in soil from the tailing pond were higher than in the other soil samples. Surprisingly, elevated heavy metal concentrations were found in sample ID 30, which was sampled in Yinchang village. There is a cinder storage near the village, and the storage is not open. After investigating, various metal elements were found to exist in the cinder [36]. The fine cinder in the wind may result in higher level of heavy metals just in a small area; metals deposited on the soil surface then gradually incorporated into the soil, thereby contributing to overall soil concentrations. Overall, the heavy metal concentrations decreased with increasing distance from the tailing pond, though there was some fluctuation of concentration. 

Based on the above analysis, there is serious heavy metal pollution of soils in the study area around the tailing pond. Cd pollution in soils was the most serious and no Cr contamination was found in the study area. This poses a potential health risk to the residents living in the study area. The elevated heavy metal concentrations in the study area resulted from continuous dispersal downstream from the tailings and waste water from tailing pond. These results coincide with several other research results, it is that high concentrations of heavy metals in soils were universal in the vicinity of mines [37,38]. 

### 3.3. Heavy Metals in Edible Parts of Vegetables and Rice

**Figure 4:**
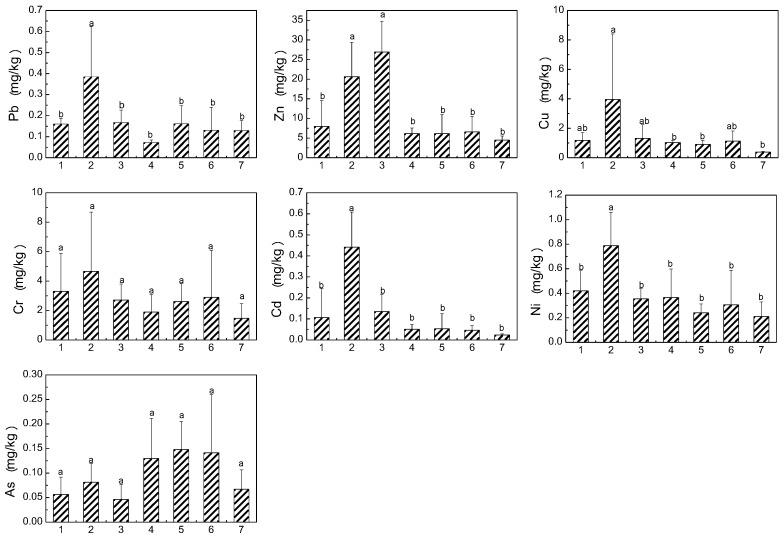
Heavy metal concentrations in seven subspecies of leafy vegetables (mean + SD) grown in the study area (*p* < 0.05). Notes: 1 garlic sprout, 2 cow soapwort, 3 cabbage, 4 Indian lettuce, 5 sweet potato leaves, 6 water spinach, and 7 scallion. Letters indicate the level of significant difference at *p* < 0.05.

**Figure 5:**
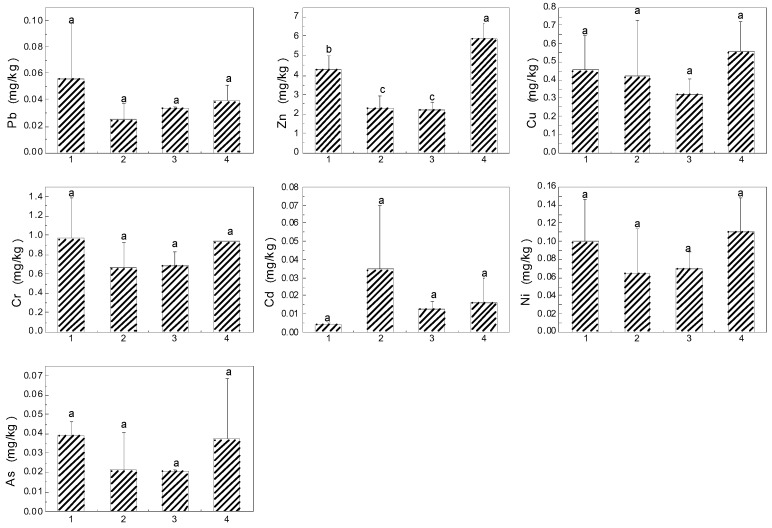
Heavy metal concentrations in four subspecies of non-leafy vegetables (mean + SD) grown in the study area (*p* < 0.05). Notes: 1 cowpea, 2 eggplant, 3 capsicum, and 4 okra. Letters indicate the level of significant difference at *p* < 0.05.

Heavy metals accumulation in edible parts of crops could have a direct impact on the health of nearby inhabitants, because crops produced in this area are mostly consumed locally. Therefore, the concentrations of heavy metals in crops could be a concern to local residents. The concentrations of heavy metals in edible parts of leafy vegetables are given in Figure 4. The heavy metal concentrations varied among different vegetables, due to their different accumulation abilities. The average concentrations of heavy metals in leafy vegetable samples decreased in the order of Zn > Cr > Cu > Ni > Pb > Cd > As. The concentrations of Pb, Cr, Cd, and As were compared with the maximum permissible level (MPL) of contaminants recommended for fresh leafy vegetables in China [39]: 0.3 mg/kg for Pb, 0.5 mg/kg for Cr, 0.2 mg/kg for Cd, and 0.5 mg/kg for As. The maximum permissible level of Ni is 0.3 mg/kg [40]. Zn and Cu are essential elements for humans, which is why the related standard in food of Zn and Cu has been abolished. Thus, we only consider the pollution of Pb, Cr, Cd, Ni, and As in vegetables. In general, the As concentrations were below the recommended values. The As concentration in leafy vegetables was minimum and far below the MPL, and this coordinated with the low bioavailability of As [41]. In leaf vegetables, the range of Pb concentration varied from 0.07 mg/kg to 0.39 mg/kg, and the average Pb content in cow soapwort was 1.3 times higher than its respective MPL. All vegetable samples exceeded the MPL for Cr, with the highest (4.7 mg/kg) in cow soapwort followed by (3.3 mg/kg) garlic sprout. Cd was only highly accumulated in cow soapwort; the concentration was 2.2 times higher than the respective MPL. 52.6% of the vegetables exceeded the permissible level of Ni, the highest concentration also being found in cow soapwort. Among the tested leafy vegetables, cow soapwort had significantly high concentrations of heavy metals except for As. Figure 5 shows the concentrations of heavy metals in edible parts of non-leafy vegetables grown in the study area. By contrast, heavy metal concentrations in non-leafy vegetables were less than that in leafy vegetables. Previous studies have shown that the transfer ability of heavy metals from soils to vegetables was stronger in leafy vegetables compared with non-leafy vegetables [4,18,42]. When compared with the MPL for vegetables, the concentrations of Pb, Cd, Ni, and As were within these recommended values, while the Cr concentration in most non-leafy vegetables exceeded the MPL for Cr. The Cr concentration in non-leafy vegetables followed the trend: cowpea> okra > capsicum > eggplant. In general, the concentrations of Pb, Zn, Cu, Cr, Ni, and As in cowpea and okra were higher than those in capsicum and eggplant. Surprisingly, eggplant accumulated high concentrations of Cd, the result being in line with survey reports that approximately 7% of eggplant in Japan contains Cd concentrations that exceed the international limit for fruiting vegetables [43]. In total, only the concentrations of Pb, Cr, Cd, and Ni were above the MPL, and the rates of excess were 3.1%, 87.5%, 9.4%, and 31.3% for Pb, Cr, Cd, and Ni, respectively. 

For the rice, the heavy metal concentrations are shown in Table 5. Zn has the highest mean concentration, while the lowest value was recorded by As, which coincided with vegetables. The mean concentrations of heavy metals in rice exceeded the maximum permissible levels (MPLs); the excess multiples were 2.2, 5.2, 2.2, 2.5, and 1.6, and the standard-exceeding rates were 100.0%, 100.0%, 80.0%, 80.0%, and 68.0% for Pb, Cr, Cd, Ni, and As, respectively. This suggested that almost all the rice sampled in the study area was severely polluted by heavy metals. Based on the above analysis, the crops planted in the vicinity of the tailing pond were affected significantly by contaminated soil environment. The heavy metal concentrations in paddy and vegetable soil decreased in the order of Zn > Pb > Cr > Cu > As > Ni > Cd, while the contents of heavy metal in rice and vegetables descended in the order of Zn > Cr > Cu > Ni > Pb > Cd > As. Even though the concentration of Cr in soil was less than related standard values, rice and vegetables were significantly contaminated by Cr. The high concentration of Cr may result from elevated Cr content in soil. The Cr limit value of 150 mg/kg has been adopted continuously since 1995, and concern still exists that this threshold may not guarantee food safety. Therefore, the Cr content in plants may still cause food safety problem. Even though the mean Pb content in paddy and vegetable soils were relatively high, the concentrations of Pb in vegetables and rice were low; this seemed to be inconsistent. Therefore, the migrating rule of Pb in soil-crop system needs to be explored. Previous studies have shown that there was no significant correlation between the Pb concentration in crops and that in soils [44,45]. Lead in soil is mainly in insoluble forms such as Pb(OH)_2_, PbCO_3_, Pb(PO_4_)_2_, and so on, while the content of soluble lead is very low. When lead enters into the soil, it exists in the form of halide and then transforms into insoluble compounds. In addition, lead can be combined with ligand to form stable metal complexes and chelates. This results in low migration of Pb from soil to crops. Therefore, lead is mainly accumulated in soil surface and it is difficult for crops to absorb it. The low As concentration in crops is the result of low bioavailability of As in soil. 

### 3.4. Health Risk Assessment

#### 3.4.1. The Daily Intake of Heavy Metals

The average daily intake of heavy metals via several exposure pathways by the local adult residents is listed in Table 6. Among all exposure pathways, we can find that the daily intake from rice diet and vegetable diet accounted for the most of total daily intake, and the daily intake of rice diet was higher than that of vegetable diet, indicating that diet was the dominant exposure route of all heavy metals. Soil ingestion was the main contributor in all soil exposure pathways, and the CDI value from soil pathways descended in the order of ingestion > dermal absorption > inhalation. Similar results were reported in previous studies [16,46].

#### 3.4.2. Health Risk Assessment

**Figure 6:**
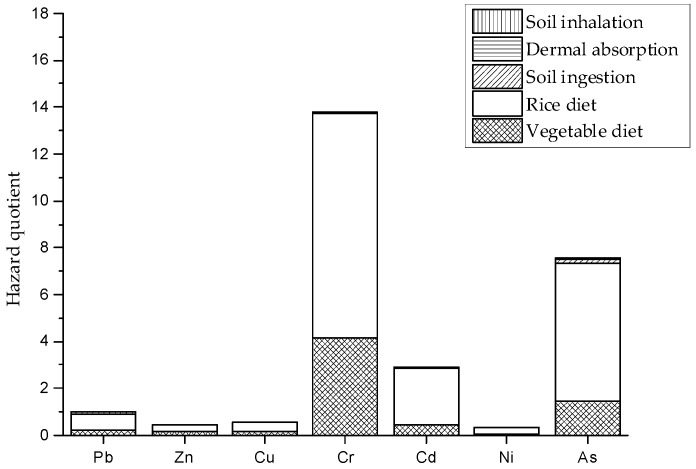
The hazard quotient of heavy metals via different pathways.

The results of no-carcinogenic risks for various heavy metals through all exposure routes are presented in Figure 6. Similar to daily intake, diet was the most significant contributor in the hazard quotient, and the HQ of rice ingestion was higher than vegetable ingestion, suggesting that rice diet presented a high risk to the health of local residents. Therefore, for the non-carcinogenic risks, we would first reduce the hazard from diet. The hazard quotient of Cr was significantly greater than other metals, primarily because of the high concentrations in vegetables and rice. The HQ values of the heavy metals decreased in the following order: Cr > As > Cd > Pb > Cu > Zn > Ni. Individually, the HQ values of Cr, As, Cd, and Pb were greater than 1 because of their high concentration or low RfD values, and the total hazard quotients of Cr, As, Cd, and Pb accounted for about 95.0% of the full HI value, indicating that the accumulation of Cr, As, Cd, and Pb would be the main cause of chronic diseases based on their high HQ values. The total non-carcinogenic hazard index (HI) value for all considered metals through multiple exposure pathways was 26.6, which is significantly higher than the safe level. This suggested that the potential health risk to local residents should be highly stressed.

For carcinogenic risk, due to the lack of carcinogenic slope factors for Pb, Zn, and Cu, only the carcinogenic risks for other four metals (As, Cd, Cr, and Ni) were estimated. Among them, the carcinogenic risk of As for all exposure modes was calculated in the model, whereas the carcinogenic risks of Cd, Cr, and Ni were considered only through inhalation. The total risk was calculated by summing the individual cancer risks across all exposure pathways. The calculated total risk (R) value was 3.4 × 10^−3^, which was higher than the acceptable range of 10^−6^ to 10^−4^. Compared to the cancer risk of Cd, Cr, and Ni, As seemed to be the predominant contaminant that created a relatively high cancer risk. The cancer risk was ranked in the order of As > Cr > Ni > Cd, illustrating that As appears to be the main pollutant source that is producing cancer among these heavy metals. Overall, the total cancer risk value was outside the acceptable range, implying great carcinogenic risk.

## 4. Conclusions

From the determination of heavy metal concentrations in water, soil, and crop samples collected from the study area, we obtained better knowledge of the impact of tailings on the environment and the potential health risk to humans. Heavy metal contamination was not found in groundwater. Concentrations of heavy metals in surface water were below the corresponding allowable levels, except for Ni and As, and the rate of excess for Ni and As was 27.8% and 5.6%, respectively. The concentrations of Zn, Cd, Ni, and As had a decreased tendency totally along the river, while the concentrations of Pb, Cu, and Cr had an irregular fluctuation along the river. Mean concentrations of Pb, Zn, Cu, Cd, Ni, and As in soil from the tailing pond were above the standard values, while the concentration of Cr was less than standard value. The heavy metal concentrations in paddy soils were higher than those in vegetables soils. The concentrations of all heavy metals in the soils exceeded the corresponding maximum allowable levels, except for Cr. For the rice soil, almost all the samples were contaminated by heavy metals. The percentage of excess of all samples decreased in the order of Cd > Zn > As > Pb > Ni > Cu > Cr, and Cd contamination in soils was the most serious. Overall, the heavy metal concentrations decreased with increasing distance from the tailing pond. Rice and vegetables were also polluted severely. Contamination of Pb, Cr, Cd, and Ni was found in leafy vegetables, and the rate of excess declined in the order of Cr > Ni > Cd> Pb. Cow soapwort accumulated the most elevated concentrations of heavy metals, except for As, among all leafy vegetables. There was just Cr contamination in non-leafy vegetables, and the Cr concentration in non-leafy vegetables followed the trend cowpea > okra > capsicum > eggplant. Heavy metal concentrations in leafy vegetables were higher than those in non-leafy vegetables due to the higher accumulating ability of heavy metals in leafy vegetables. For rice samples, almost all samples were contaminated by all the heavy metals, and Cr pollution was the worst; these results are similar to the results for vegetables. This indicated that rice and vegetables planted in the area around the tailing pond have been seriously contaminated by heavy metals. Among all the exposure pathways, diet was the main contributor in the hazard quotient. HQ values of Cr, As, Cd, and Pb were more than the allowable levels, and Cr has the biggest carcinogenic effect. The total hazard index value was 26.6, which considerably exceeded the allowable levels. As generates the greatest cancer risk, and the total cancer risk of As, Cr, Cd, and Ni was 3.4 × 10^−3^, which exceeded the allowable range.

Overall, results suggested that surrounding sites of the tailing pond are highly polluted, and more attention should be paid to the potential health risks of heavy metals to residents in the vicinity of the tailing pond. The following measures can be taken: (1) prevent the transfer of heavy metals in tailing pond into the surrounding environment; (2) soil remediation needs to be effected immediately; (3) local residents should avoid planting rice and leaf vegetables; and (4) residents should carefully select their food source to avoid consumption of local agricultural products polluted by tailing. However, there are some differences in the physical characteristics and living habits of people at home and abroad, and some of the exposure parameters are based on foreign data, which inevitably brings some uncertainty to the evaluation results. In order to reduce the heavy metal concentrations in the tailing pond and the health risk to residents living in the surrounding environment, we plan to remediate the tailing pond by planting plants with high enrichment ability for heavy metals in the future. 

## Figures and Tables

**Table 1:** Equations of daily intake dose via various exposure pathways.

Exposure Pathway	Exposure Calculations
ingestion of soil	${\mathrm{CDI}}_{\mathrm{ingestion-soil}}=\frac{\mathrm{CS}\times \mathrm{IR}\times \mathrm{CF}\times \mathrm{EF}\times \mathrm{ED}}{\mathrm{BW}\times \mathrm{AT}}$
inhalation of soil	${\mathrm{CDI}}_{\mathrm{inhale-soil}}=\frac{\mathrm{CS}\times {\mathrm{PM}}_{10}\times \mathrm{DAIR}\times \mathrm{PIAF}\times \mathrm{FSPO}\times \mathrm{CF}\times \mathrm{EF}\times \mathrm{ED}}{\mathrm{BW}\times \mathrm{AT}}$
dermal absorption of soil	${\mathrm{CDI}}_{\mathrm{dermal-soil}}=\frac{\mathrm{CS}\times \mathrm{AF}\times \mathrm{SA}\times \mathrm{ABS}\times \mathrm{CF}\times \mathrm{EF}\times \mathrm{ED}}{\mathrm{BW}\times \mathrm{AT}}$
oral intake of crop	${\mathrm{CDI}}_{\mathrm{crop}}=\frac{C_{\mathrm{crop}}\times {\mathrm{IR}}_{\mathrm{crop}}\times \mathrm{EF}\times \mathrm{ED}}{\mathrm{BW}\times \mathrm{AT}}$

CDI: chronic daily intake dose; CS: Heavy metal content in soil (mg/kg); IR: Soil ingestion rate (mg/day); CF: Conversion factor (kg/mg); EF: Exposure frequency (day/a); ED: Exposure duration (a); BW: Body weight (kg); AT: Average time (day); PM_10_: Content of inhalable particulates in ambient air (mg/m^3^); DAIR: Daily air inhalation rate (m^3^/day); PIAF: Retention fraction of inhaled particulates in body; FSPO: Fraction of soil-borne particulates in air; AF: Skin adherence factor (mg/cm^2^); SA: Exposed surface area of skin (cm^2^); ABS: Dermal absorption factor; C_crop_: Heavy metal content in crops (mg/kg); IR_crop_: Ingestion rate (g/day).

**Table 2:** The definition and value of exposure parameters.

Parameter	Definition	Value of Parameter	Reference
CS	Heavy metal content in soil (mg/kg)	observed value	-
IR	Soil ingestion rate (mg/day)	100	[21]
CF	Conversion factor (kg/mg)	10^−6^	[22]
EF	Exposure frequency (day/a)	350	[22]
ED	Exposure duration (a)	30	[22]
BW	Body weight (kg)	60.6	[23]
AT	Average time (day)	365 × 70 (carcinogens) 365 × ED (non-carcinogens)	[21]
PM_10_	Content of inhalable particulates in ambient air (mg/m^3^)	0.15	[24]
DAIR	Daily air inhalation rate (m^3^/day)	14.5	[24]
PIAF	Retention fraction of inhaled particulates in body	0.75	[24]
FSPO	Fraction of soil-borne particulates in air	0.5	[24]
AF	Skin adherence factor (mg/cm^2^)	0.2	[21]
SA	Exposed surface area of skin (cm^2^)	5408	[21]
ABS	Dermal absorption factor	0.001	[21]
C_crop_	Heavy metal content in crops (mg/kg)	observed value	-
IR_crop_	Ingestion rate (g/day)	402 (vegetables) 348.44 (rice)	[23]

**Table 3:** Heavy metal concentration in water samples (μg/L).

Water		pH	Heavy Metal Concentration (μg/L)						
			Pb	Zn	Cu	Cr	Cd	Ni	As
surface water	Mean ± stdev.	7.50 ± 0.34	4.33 ± 2.54	269.90 ± 269.52	2.40 ± 1.22	1.69 ± 1.10	1.04 ± 1.27	11.40 ± 13.72	24.62 ± 46.54
	range	(6.97–7.81)	(0.82–10.93)	(20.24–969.74)	(0.30–6.15)	(0.83–4.95)	(0.04–4.04)	(1.11–45.31)	(2.13–188.25)
groundwater	mean ± stdev.	6.22 ± 0.50	7.71 ± 10.62	102.66 ± 80.32	13.63 ± 17.26	3.69 ± 3.73	0.45 ± 0.32	3.56 ± 3.43	1.36 ± 1.66
	range	(5.41–7.04)	(0.56–31.26)	(24.89–263.77)	(0.26–39.77)	(0.57–11.08)	(0.03–0.92)	(0.70–10.55)	(0.03–4.70)
Grad V ^1^		6.0–9.0	100	2000	1000	100	10	20	100
Grad III ^2^		6.5–8.5	50	1000	1000	50	10	50	50

Notes: ^1^ Grad V of Environmental Quality Standards for Surface Water (GB3838-2002); ^2^ Grad III of Environmental Quality Standards for Groundwater (GB/T14848-93).

**Table 4:** Heavy metal concentrations in soils (mg/kg).

Soil Type	Heavy Metals	Pb	Zn	Cu	Cr	Cd	Ni	As
Tailing	Mean ± stdev.	6349.7 ± 724.1	1552.0 ± 530.6	206.3 ± 126.6	140.2 ± 55.7	11.4 ± 7.8	117.2 ± 49.1	3021.2 ± 298.7
	Median	2409.8	1432.1	143.1	130.1	14.5	140.4	3146.5
	Range	1932.6–8340.6	1091.7–2132.4	123.7–352.1	90.2–200.2	2.6–17.1	60.9–150.4	2680.3–3236.9
Paddy soil	Mean ± stdev.	245.6 ± 200.1	491.0 ± 319.7	35.6 ± 23.4	59.8 ± 19.4	2.6 ± 1.6	37.1 ± 29.7	54.8 ± 32.8
	Median	154.5	406.1	27.2	57.9	2.3	28.7	44.9
	Range	59.6–992.6	70.4–1362.2	10.5–101.5	29.7–97.7	0.6–7.5	3.1–135.4	10.5–156.6
Vegetable soil	Mean ± stdev.	138.2 ± 210.4	321.8 ± 547.6	27.4 ± 19.7	30.5 ± 17.1	1.8 ± 3.2	11.7 ± 10.9	25.2 ± 33.4
	Median	79.2	184.0	21.4	23.3	0.9	7.2	19.6
	Range	29.9–1119.0	45.9–2847.5	9.8–98.0	12.3–77.3	0.1–16.7	3.5–55.6	6.4–189.9

**Table 5:** Metal concentrations in edible parts of rice (mg/kg).

Metals		Pb	Zn	Cu	Cr	Cd	Ni	As
rice	mean	0.45	17.34	2.84	5.21	0.44	1.00	0.32
	stdev.	0.21	5.38	1.17	3.04	0.34	1.17	0.15
	min	0.23	8.94	1.11	0.01	0.02	0.18	0.13
	max	1.14	32.10	5.73	12.67	1.39	4.96	0.75
standard value ^1^		0.2	-	-	1.0	0.2	0.4	0.5

^1^ Maximum level of contaminants in foods (GB2762-2005).

**Table 6:** Daily intake from heavy metals via various exposure pathways (µg/kg/day).

Pathway	Pb	Zn	Cu	Cr	Cd	Ni	As
Soil ingestion	2.93 × 10^−1^	6.23 × 10^−1^	4.88 × 10^−2^	6.98 × 10^−2^	3.30 × 10^−3^	3.72 × 10^−2^	6.11 × 10^−2^
Soil dermal absorption	3.17 × 10^−3^	6.73 × 10^−3^	5.28 × 10^−4^	7.55 × 10^−4^	3.57 × 10^−5^	4.02 × 10^−4^	6.60 × 10^−4^
Soil inhalation	2.39 × 10^−3^	5.08 × 10^−3^	3.98 × 10^−4^	5.69 × 10^−4^	2.69 × 10^−5^	3.03 × 10^−4^	4.98 × 10^−4^
Vegetable diet	0.72	46.5	5.99	12.5	0.46	1.59	0.43
Rice diet	2.47	95.6	15.7	28.7	2.42	5.50	1.77
